# Relationship Between the Free and Total Methotrexate Plasma Concentration in Children and Application to Predict the Toxicity of HD-MTX

**DOI:** 10.3389/fphar.2021.636975

**Published:** 2021-04-29

**Authors:** Wei-Chong Dong, Jia-Liang Guo, Xi-Kun Wu, Meng-Qiang Zhao, Hao-Ran Li, Zhi-Qing Zhang, Ye Jiang

**Affiliations:** ^1^ Department of Pharmaceutical Analysis, School of Pharmacy, Hebei Medical University, Shijiazhuang, China; ^2^ Department of Pharmacy, The Second Hospital of Hebei Medical University, Shijiazhuang, China; ^3^ Department of Orthopaedics, The Third Hospital of Hebei Medical University, Shijiazhuang, China

**Keywords:** methotrexate, free concentration, total concentration, HFCF-UF, nephrotoxicity

## Abstract

High-dose methotrexate (HD-MTX) can be highly effective as well as extremely toxic. Many drug molecules can bind to plasma proteins to different extents *in vivo*, whereas only the free drug can reach the site of action to exert a pharmacological effect and cause toxicity. However, free MTX concentrations in plasma have not been reported. Traditional analyses of free drugs are both cumbersome and inaccurate. We collected 92 plasma samples from 52 children diagnosed with ALL or NHL or other lymphomas that were treated with HD-MTX. The hollow fiber centrifugal ultrafiltration (HFCF-UF) was used to prepare plasma samples for analysis of the free MTX concentration. Protein precipitation was employed to measure the total MTX concentration. The HFCF-UF is a simple method involving a step of ordinary centrifugation; the validation parameters for the methodological results were satisfactory and fell within the acceptance criteria. A linearity coefficient *r* ^2^ of 0.910 was obtained for the correlation between the free and total MTX plasma concentrations in 92 plasma samples. However, the free and total MTX concentrations was only weakly correlated in 16 clinical plasma specimens with total MTX concentrations >2 μmol L^−1^ (*r* ^2^ = 0.760). Both the free and total MTX concentrations at 42 h were negatively correlated with the creatinine clearance (CCr) level (*P* = 0.023, *r* = −0.236 for total MTX and *P* = 0.020, *r* = −0.241for free MTX, respectively). The free MTX concentration could not be accurately estimated from the total MTX concentration for patients with high MTX levels which are conditions under which toxic reactions are more likely to occur. High plasma MTX levels could become a predictor of the occurrence of MTX nephrotoxicity to draw people's attention. The proposed HFCF-UF method is a simple and accurate way to evaluate efficacy and toxicity in clinical therapeutic drug monitoring.

## Introduction

Methotrexate (MTX) is an antimetabolite for folic acid (Bluett et al., 2019). MTX is an essential component of therapy for acute lymphoblastic leukemia (ALL) and is active against many types of cancer; however, MTX use needs to be monitored for potential side effects, such as bone marrow suppression, alopecia, stomatitis and the development of hepatic fibrosis or cirrhosis (De Abreu et al., 2015; Karami, et al., 2019). MTX doses of 500 mg/m^2^ or higher given intravenously are defined as high-dose methotrexate (HD-MTX) (Howard et al., 2016). High doses are often more effective than lower doses but can cause significant toxicity, including acute kidney injury (AKI), which not only leads to morbidity and occasional mortality but may also interrupt cancer treatment (Christensen et al., 2012; Taylor et al., 2020). Thus, MTX must be administered with rigorous standardized supportive care to prevent unacceptable toxicity (Roberts et al., 2016; Ramsey et al., 2018).

The measurement of MTX levels is recommended as a routine practice. Prolonged exposure to toxic methotrexate concentrations without timely recognition and treatment can lead to significant morbidity and mortality, especially for patients with delayed methotrexate excretion (Skarby et al., 2003; Howard et al., 2016; Roberts et al., 2016; Svahn et al., 2017; Ramsey et al., 2018). Therefore, accurate monitoring of MTX concentrations is extremely important to accurately assess patient excretion and reduce the incidence of toxic reactions.

It is well known that many drug molecules can bind to plasma proteins to different extents *in vivo*, whereas only an unbound drug can reach the site of action to exert a pharmacological effect and cause toxicity (Dasgupta, 2007; Zhang et al., 2012). Therefore, measuring the free drug concentration in clinical plasma or serum samples, rather than the total concentration, is often considered to be more salient for therapeutic drug monitoring (TDM) (Berthoin et al., 2009; Zhang et al., 2015). To the best of our knowledge, TDM of MTX is currently based mainly on total MTX concentration measurements. Free MTX measurements and correlations between free and total MTX plasma concentrations have not been reported.

Renal function has been correlated with the MTX concentration, and high MTX concentrations can predict the occurrence of MTX-related renal toxicity in adults (Yang et al., 2018). However, the relationship between the MTX plasma concentration and nephrotoxicity in children remains controversial. The MTX concentration in children has been negatively correlated with the CCr level, as for adults (Hempel et al., 2003). However, other studies have shown no relationship between the MTX concentration and CCr in children (Evans et al., 1984; Joannon et al., 2004). All the above mentioned studies were based on the total MTX concentration. There are no reports on the relationship between the free MTX concentration and MTX nephrotoxicity, although the free drug concentration is the most direct cause of toxicity.

Commonly used methods for free drug analysis are equilibrium dialysis (ED) and centrifugal ultrafiltration (CF-UF) (Rakhila et al., 2011; Saari et al., 2012). However, ED is cumbersome and time-consuming; the addition of a dialysis buffer solution can change the true plasma condition and result in poor sensitivity of the method (Herforth et al., 2002; Rakhila et al., 2011). Recently, CF-UF devices have been widely used for separating a free drug from human plasma. However, the ultrafiltrate volume has been demonstrated to be large and poorly controlled, which can disturb the initial drug-protein binding equilibrium; in addition, an experimenter must be well trained to prevent poor accuracy and precision (Zhang and Musson, 2006; Dong et al., 2013b). These problems may limit the application of free drug analysis in clinical TDM.

In this study, we developed a hollow fiber centrifugal ultrafiltration (HFCF-UF) technology to determine the free MTX concentration in human plasma. The ultrafiltrate can be easily controlled to a small and invariant volume using this method. The proposed procedure did not disturb the drug-protein binding equilibrium, producing an accurate and precise result. The newly developed method was successfully conducted in 92 plasma sample from children with ALL, NHL or other lymphoma that were treated with HD-MTX. Correlations were determined between the free and total MTX concentrations in human plasma, as well between the free and total MTX concentrations and renal function.

## Experimental

### Chemicals and Materials

Methotrexate standard (No.100138–201606) was purchased from the National Institutes for Food and Drug Control (Beijing, China). Tinidazole standard (No. 12060343) was obtained from Shijiazhuang No.4 Pharmaceutical Co. LTD (Shijiazhuang, China). The blank human serum was offered from the Second Hospital of Hebei Medical University. Methanol (HPLC-grade) was purchased from Fisher Chemical (Lake Forest, CA). The deionized water was prepared by a Milli-Q50 water purification system (Millipore, Bedford, MA). All the chemicals used were of analytical grade.

The HFCF-UF device were obtained from Hebei Heping Medical Equipment Factory (shijiazhuang, China). The molecular cut-off was 10 kDa. The wall thickness of this fiber was 150 μm and the inner diameter was 1,000 μm. The slim glass tubes (7 cm of height and 3.5 mm of inner diameter).

### Apparatus and Instruments

An ACQUITY UPLC H-Class (Waters, United States) was used for analysis. Data were collected and analyzed using Empower 3 (Waters, United States). The R18 centrifuge Baiyang (Beijing, China) and CPA225D electronic analytical balance (Germany, Sartorius) were used. XW-80 Vortex mixer (Shanghai medical university Instrument Co., Shanghai, China) and QGC-12T Nitrogen blowing instrument (Quandao. Corp, Shanghai, China) were also employed here.

### UPLC Conditions

MTX was separated using a Waters BEH C_18_ column (50 mm × 2.1 mm, 1.7 μm). The mobile phase consisted of methanol and a 0.05 M phosphate buffer (pH 6.2) (17:83, v/v) at a flow rate of 0.2 ml min^−1^. The column temperature was maintained at 30°C. The detected wavelength was 302 nm, and the injection volume was 2 μL.

### Standard Solution and Quality Control Samples

A stock MTX solution was prepared in methanol at a concentration of 500 μmol L^−1^ and stored at 4°C. A series of working solutions were prepared by diluting the stock solution with deionized water to final concentrations of 100, 50, 20, 10, 5, 2, 1, and 0.5 μmol L^−1^. A stock solution of the internal standard tinidazole was prepared in methanol at a concentration of 250 μg mL^−1^. The stock solution was diluted with deionized water to prepare 25 μg mL^−1^ internal working solution.

Calibration standards for the analysis of the free MTX concentration of 10, 5, 2, 1, 0.5, 0.2, 0.1, and 0.05 μmol·L^−1^were prepared in a blank human plasma ultrafiltrate (450 μL) spiked with 25 μL of MTX standard working solutions and 25 μL of internal solutions (25 μg mL^−1^). The QC samples were prepared at concentrations of 0.05, 1, and 8 μmol L^−1^.

### Sample Preparation for Determination of Free Methotrexate Concentration by Hollow Fiber Centrifugal Ultrafiltration

**FIGURE 1:**
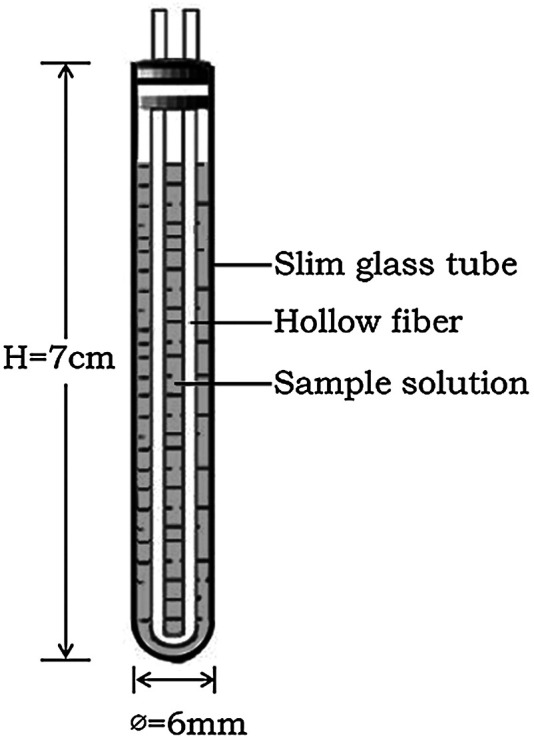
Hollow fiber centrifugal ultrafiltration device (HFCF–UF).

A hollow fiber was cut into 15-cm segments, sonicated in methanol and allowed to dry naturally until use. Approximately 500 μL of a plasma sample were placed in a slim glass tube. The hollow fiber (15 cm) was bent into a U-shape and inserted into the slim glass tube, as shown in Figure 1. After ultrafiltration for 10 min at 2.4 × 10^3^ g, a syringe was used to push the ultrafiltrate in the lumen of the hollow fiber out through the other end of the hollow fiber, and 2 μL of the ultrafiltrate were injected into the UPLC for analysis.

### Sample Preparation for Determination of Total Methotrexate Concentration

Approximately 200 μL of a plasma sample were added to 500 μL of acetonitrile in a 1.5-ml centrifuge tube. The mixture was homogenized for 2 min using a vortex mixer and then centrifuged at 1.0 × 10^4^ g for 5 min. A volume of 500 μL of the supernatant was transferred to a clean centrifuge tube and subsequently evaporated by nitrogen at 50°C. The residue was reconstituted using 0.2 ml of deionized water, and 1.5 μL of the resulting mixture was injected into the UPLC.

### Sample Collection

The study protocol was approved by the Ethics Committee of The Second Hospital of Hebei Medical University. A total of 92 plasma samples were obtained from 52 children with acute lymphoblastic leukemia (ALL) or non-Hodgkin lymphoma (NHL) or other lymphoma from August 2019 to August 2020. According to our guideline for the diagnosis and treatment of acute lymphoblastic leukemia or lymphoma in children. All the children were administered high-dose methotrexate (HD-MTX) at 2–5 g/m^2^. The MTX infusion was followed with leucovorin (LV) rescue at the beginning dose of 15 mg/m^2^/time, 3–8 times, once 6 h. The MTX plasma concentration was monitored at 42 h and the dose of LV rescue was adjusted according to the MTX concentration. The rescue was performed every 6 h until the concentration was lower than 0.25 μmol L^−1^. Approximately 3 ml of blood were collected in a centrifuge tube 42 h after the MTX infusion and centrifuged for 5 min at 6.0 × 10^3^ g; the obtained plasma samples were then immediately analyzed or stored at −80°C for testing.

### Statistical Analysis

SPSS 22.0 software was used to perform a statistical analysis. A regression analysis was used to correlate the free and total MTX plasma concentrations. The Spearman correlation coefficient was used to estimate the correlation between the creatinine clearance (CCr) and the plasma MTX concentration. The data assessments were double-tailed, and values of *P* < 0.05 were considered to be statistically significant.

## Results

### Nonspecific Binding

The difficulty of validating a method to determine the free drug concentration is generally well recognized, because a plasma sample with a known free drug concentration is not available. An analyst performing ultrafiltration must bear in mind that the major disadvantage of this procedure is the nonspecific binding (NSB) of drugs to filter membranes or glass and plastic devices: therefore, the NSB must be quantified (Li et al., 2011). Three different hollow fiber materials, including polysulfone, polyvinylidene difluoride and polyacrylonitrile, were chosen to determine the NSB. The ratios of the concentrations obtained from polyacrylonitrile HFCF-UF to the corresponding standard ultrafiltrate concentrations were approximately 100% for three different MTX concentrations (8, 1 and 0.05 μmol L^−1^) and the internal standard solutions (25 μg mL^−1^ tinidazole). Thus, polyacrylonitrile HFCF-UF had an insignificant NSB and was used in subsequent experiments. The corresponding ratios for polysulfone and polyvinylidene difluoride HFCF-UF for MTX were approximately 25 and 50%, respectively, which suggested a nonnegligible NSB for these materials and precluded the directly use of these materials in subsequent experiments.

### Method Validation

#### Selectivity

**FIGURE 2:**
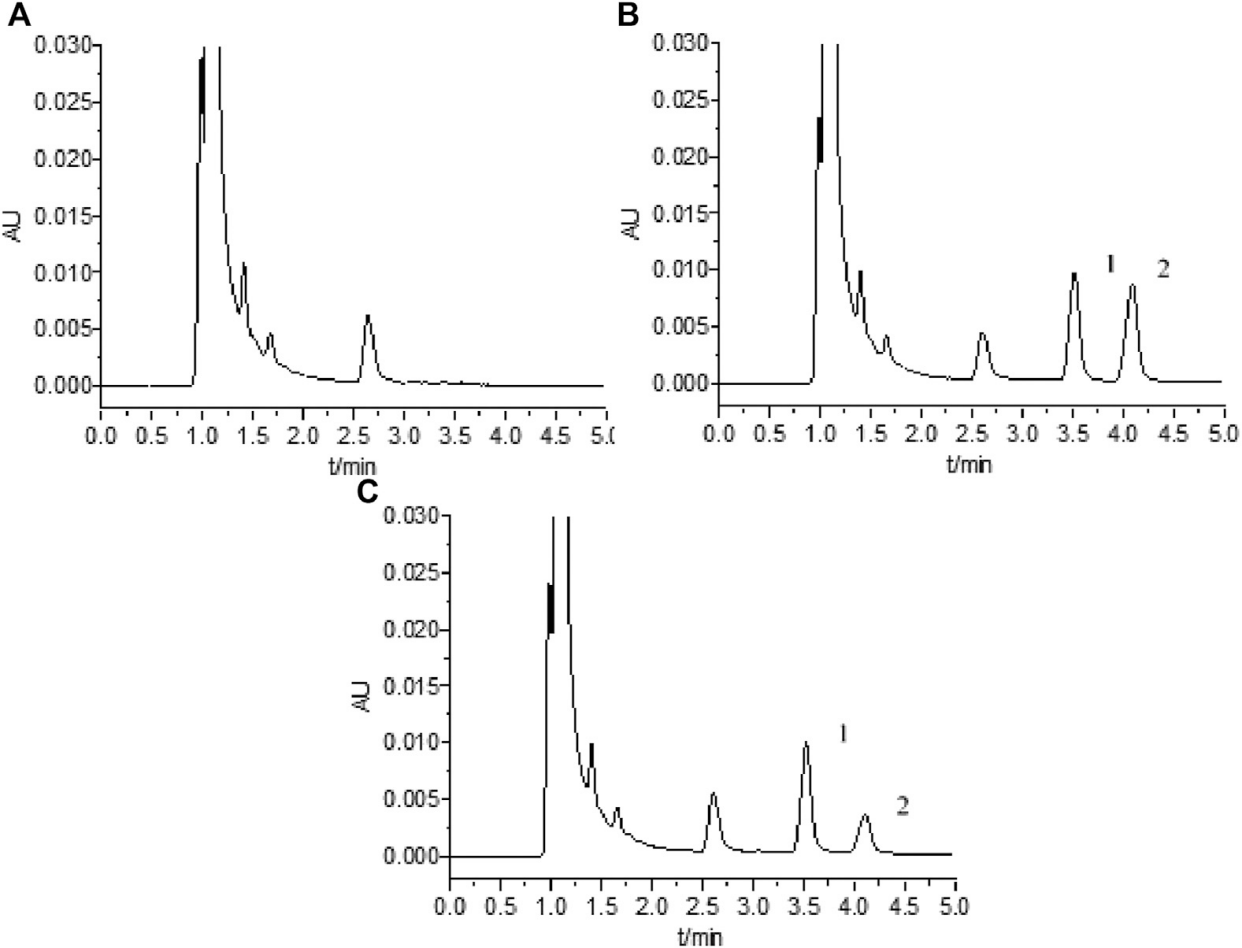
UPLC chromatograms for MTX: **(A)** blank plasma; **(B)** blank plasma spiked with 2 μmol L^−1^ MTX and 2.5 μg mL^−1^ internal standard; **(C)** clinical plasma sample; 1 and 2 indicate peaks for internal standard and MTX, respectively.

We analyzed the blank plasma ultrafiltrate, QC samples (a blank plasma ultrafiltrate standard solution spiked with 2 μmol L^−1^ MTX and a 2.5 μg mL^−1^ internal standard) and the clinical plasma sample. The UPLC chromatograms (Figures 2A–C) indicated that the method was sufficiently specific.

#### Linearity, Limit of Detection and Limit of Quantification

An assay calibration curve was generated by plotting the peak area ratios of MTX and the internal standard at eight different MTX concentrations (10, 5, 2, 1, 0.5, 0.2, 0.1, and 0.05 μmol L^−1^) for linear regression analysis. The linear calibration result for free MTX was *A* = 0.297*X* + 0.001, with a correlation coefficient of 0.999 using a weighted factor 1/*C* ^2^. The limit of detection (LOD) and the limit of quantification (LOQ) were 0.0125 and 0.05 μmol L^−1^, respectively.

#### Accuracy, Precision, and Absolute Recovery

The intraday precision and accuracy were determined using five replicates of QC samples (0.05, 1, and 8 μmol L^−1^) on the same day. The interday precision was evaluated on 3 consecutive days. The absolute recovery was evaluated in terms of the ratio of the peak areas of the QC samples to those of the standard solution with the same concentration for six replicates. Satisfactory results were obtained, as shown in Table 1.

**TABLE 1:** Results of recovery and precisions test for the analysis of free MTX in human plasma (*n* = 5).

Added concentration (μmol·L^−1^)	Found concentration (μmol·L^−1^)	Relative Recovery (%)	Intra RSD (%)	Inter- RSD (%)	Absolute Recovery (%)
0.05	0.048	95.99	3.92	6.50	92.9
1	0.95	95.76	1.16	1.66	84.1
8	8.04	100.5	0.45	1.50	90.8

The average recovery and the absolute recovery at three different concentrations were 84.1–100.5%, and both the intra- and interday precisions (RSD) were less than 7%. The absolute recovery of the internal standard (2.5 μg mL^−1^) was 97.3%.

#### Stability and Dilution Effect

The stability of plasma samples is crucial for to ensuring that the analyte concentration is not affected by the sample preparation, sample analysis or storage conditions. We evaluated the room temperature stability, freezing stability, freeze-thaw stability and postprocessing stability of the samples. The results showed that all the QC samples (0.05, 1, and 8 μmol L^−1^) were stable with RSD values below 10% under the following conditions: maintenance at room temperature (25°C) for 12 h, three freeze-thaw cycles, maintenance at ^−^80°C for 21°days and for 8 h after processing at room temperature.

We evaluated the dilution effect of the samples with QC samples at 0.05, 1, and 8 μmol L^−1^. These sample (*n* = 5) were diluted at 10 times or 100 times with blank plasma ultrafiltrate from our prepared spiked samples at high concentration of 5, 100, and 80 μmol L^−1^, respectively. The average accuracy at three different concentrations were 101, 95.3, and 104%.

### Application to Clinical Samples

A volume of 500 μL of the plasma samples was placed in the HFCF-UF apparatus to determine the MTX free concentration, and 200 μL of plasma were used to determine the total MTX concentration, following the procedures described in *Sample preparation for determination of free MTX concentration by HFCF-UF* and *Sample preparation for determination of total MTX concentration*, respectively. If the MTX concentration was larger than 10 µM, we will dilute ultrafiltrate samples (10 times or 100 times) to our concentration range with blank plasma ultrafiltrate and reanalysis by our developed method. It is recommended that the MTX infusion be followed with leucovorin (LV) rescue, at initial dose of 15 mg/m^2^/time, once 6 h. Then the rescue dosage depends on the MTX concentration for a total MTX plasma concentration (De Abreu et al., 2015; Howard et al., 2016). The sample information and corresponding results are presented in Table 2.

**TABLE 2:** The TDM results of MTX in 92 patients with ALL or other lymphoma.

Characteristic	mean ± SD, (range)
Gender	63 (male): 29 (female)
Age (years)	6.72 ± 4.10 (1–16)
Body weight (kg)	27.85 ± 16.77 (11.1–80.0)
Body height (cm)	121.65 ± 28.62 (60–180)
Dose (g/m^2^)	3.75 ± 0.96 (1.93–5.27)
Cr (μmol/L)	31.39 ± 14.07 (12.00–86.00)
CCr (ml/min), 42 h	138.83 ± 55.95 (41.59–316.32)
Total concentration (μmol·L^−1^)	1.47 ± 2.11 (0.16–11.19)
Free concentration (μmol·L^−1^)	0.79 ± 1.20 (0.07–6.15)
Plasma protein binding rate (%)	48.54 ± 14.69 (16.57–89.99)
Albumin concentration (μmol/L)	31.39 ± 14.01 (21.20–52.80)

**FIGURE 3:**
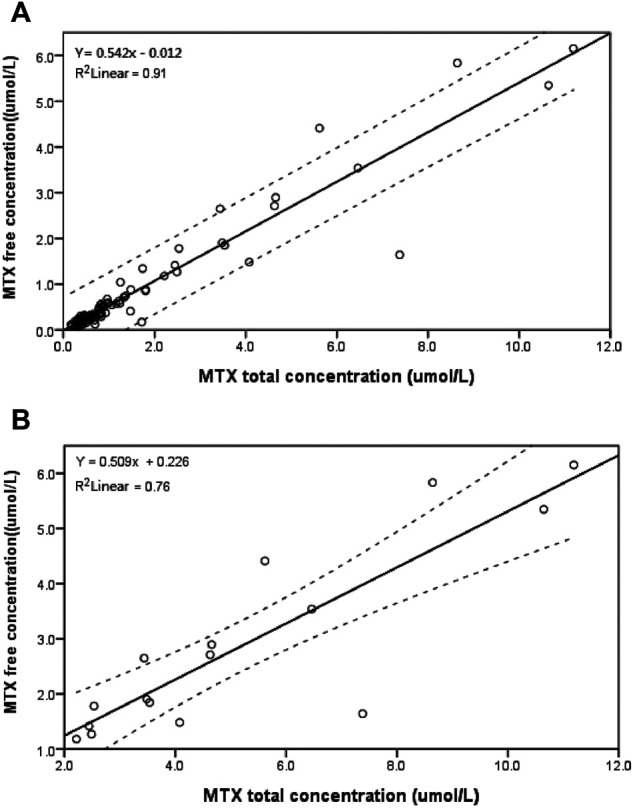
Relationship between free and total MTX concentrations for **(A)**: 92 clinical plasma samples from children; **(B)**: 16 clinical plasma samples with MTX total concentration >2 μmol L^−1^ at 42 h from children; dotted lines show 95% confidence interval.

**FIGURE 4:**
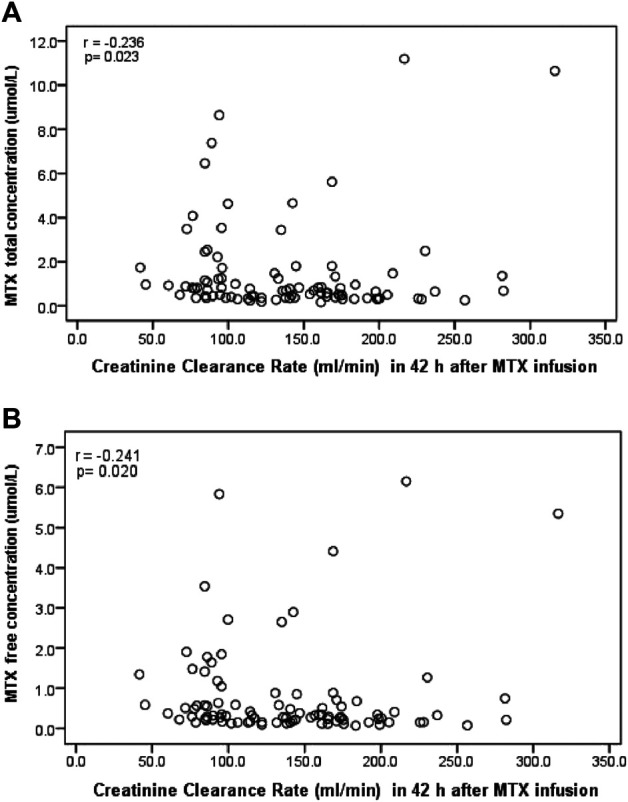
Spearman’s rank correlation analysis of MTX concentration and CCr level 42 h after MTX infusion for **(A)**: total MTX concentration and **(B)**: free MTX concentration.

Figure 3A shows the relationship between the free and total MTX plasma concentrations for 92 plasma samples (*r* ^2^ = 0.910). We also correlated the free and total MTX plasma concentrations for 16 clinical plasma samples with total MTX concentrations >2 μmol L^−1^ (*r* ^2^ = 0.760): the results are shown in Figure 3B. The results of the Spearman’s rank correlation for the creatinine clearance (CCr) with the free and total MTX concentrations in the plasma are shown in Figure 4. The total MTX concentration at 42 h was negatively correlated with the CCr level (Figure 4A) (*P* = 0.023, *r* = -0.236). The free MTX concentration at 42 h was also negatively correlated with the CCr level (Figure 4B) (*P* = 0.020, *r* = -0.241).

## Discussions

### Comparison of Hollow Fiber Centrifugal Ultrafiltration and Commercial Centrifugal Ultrafiltration

Commercial centrifugal ultrafiltration (CF-UF) has been widely used in recent years to measure free drug concentrations (Zhang and Musson, 2006; Berthoin et al., 2009; Li et al., 2011; Saari et al., 2012; Kolmer et al., 2017). This method is simple and fast but also has disadvantages. A portion of the membrane used in CF-UF lies perpendicular to the centrifugal force. Thus, the membrane can be affected by the molecular sieve and concentration polarization (Zhang and Musson, 2006; Li et al., 2011; Dong et al., 2013b). It has been reported that the ratio of the ultrafiltrate volume to the sample solution cannot be well controlled using CF-UF, which typically results in a large ultrafiltrate yield. Consequently, the protein-binding equilibrium may be disturbed, producing inaccurate and imprecize results. The experimenter must be well trained to ensure the accuracy of the results (Dong et al., 2013a; Zhang et al., 2013a).

In this study, a HFCF-UF method was developed to determine the free MTX plasma concentration: accurate and precise results were obtained, and the time and workload required for sample preparation procedure were reduced, because the simple proposed procedure involves a step of ordinary centrifugation that can be easily performed in any laboratory (Dong et al., 2013a; Dong et al., 2013b; Zhang et al., 2013b; Zhang et al., 2014; Zhang et al., 2015). In the proposed method, the membrane was completely parallel to the centrifugal force, and small molecules could freely pass through the membrane without being affected by molecular sieves and concentration polarization (Li et al., 2010; Zhang et al., 2013b). The volume ratio of the ultrafiltrate to the sample solution was sufficiently small and constant to minimally affect the protein-binding equilibrium (Dong et al., 2013b). Furthermore, the volume ratio of the ultrafiltrate to the sample solution could be well controlled. Therefore, satisfactory validation parameters were obtained for the methodological results (Dong et al., 2013a; Kolmer et al., 2017). The HFCF-UF procedure has also been shown to improve biosafety, which may become increasingly important for medical staff with the COVID-19 outbreak (Wang et al., 2017; Dong et al., 2020).

### Data Analysis of Relationship Between Free and Total Methotrexate Concentrations

It is well known that many drugs bind to plasma proteins. Only the free drug fraction is pharmacologically active (Dasgupta, 2007; Zhang et al., 2012). However, TDM is usually performed by determining the total drug concentration. The free drug concentration can be estimated from the total drug concentration in the plasma of a normal patient with a stable plasma-protein binding rate. However, there is a poor correlation between the total and free drug concentrations for plasmas from patients with serious infections or severe complications, such as uremia, liver disease and hypoalbuminemia or who are receiving concurrent medications and for plasmas with nonlinear protein binding characteristics (Berthoin et al., 2009; Zhang et al., 2013b; Zhang et al., 2015; Kolmer et al., 2017). Thus, treatment failure or drug toxicity can result, even if the total drug concentration is within the normal therapeutic range. Therefore, it is important and highly recommended that the free drug concentration be monitored.

Figure 3A shows a linear relationship between the free and total MTX concentration for 92 plasma samples. However, most of the samples were obtained from children characterized as having rapid body metabolism. Thus, further study and verification is required to determine whether the same result would be obtained for children with delayed MTX elimination or in adults, elderly patients or patients with severe complications.

Figure 3B shows a weak correlation between the free and total MTX concentrations in 16 clinical plasma samples where the total MTX concentration >2 μmol L^−1^ at 42 h (*r* ^2^ = 0.760). This result shows that the total MTX concentration is not a good predictor of the free MTX concentration. It has reported that MTX concentrations >1 μmol L^−1^ at approximately 48 h or >0.1 μmol L^−1^ at 72 h are defined as MTX elimination delay (Yang et al., 2018). It also reported that the MTX of concentration at 36h > 3 μmol L^−1^ developed delayed elimination (Skarby et al., 2003). We choose 2 μmol L^−1^ as the cut-off value in our present work to consider that the 16 samples with total MTX concentration >2 μmol L^−1^ at 42 h are from patients with delayed elimination. High MTX levels in children, because of delayed MTX elimination, for example, are likely to induce toxic reactions; thus, accurate monitoring of the free MTX concentration is required.

In Table 2, the average protein binding ratio of MTX is 48.56%, which is in agreement with values reported in the literature (35–50%) (Jouyban et al., 2011). However, the standard deviation in the protein binding ratio was 14.69 with a large range of 16.57–89.99%. This result further demonstrates the necessity of monitoring the free drug concentration to accurately evaluate efficacy and toxicity.

MTX is albumin-binding Wang et al. (2017): however, whether an unusually high free MTX concentration could be induced in a patient with hypoalbuminemia has been not investigated in this study. The plasma albumin level in the 92 plasma samples in our study was almost normal, and only 5 samples exhibited a low albumin level. Therefore, more clinical samples from patients with hypoalbuminemia or *in vitro* tests need to be investigated in the future.

### Data Analysis of Correlation Between Creatinine Clearance Level and Methotrexate Concentration

The kidneys provide the main pathway for MTX elimination, and approximately 70–90% of unchanged MTX is excreted in urine (Yang et al., 2018). HD-MTX are often more easily to cause drug accumulation to induce nephrotoxicity (Howard et al., 2016; Ramsey et al., 2018; Yang et al., 2018). A negative correlation between kidney function and the MTX concentration has been reported for adults, and high MTX concentrations during HD-MTX treatment in Chinese adults with ALL or NHL is a predictor of the occurrence of renal toxicity (Yang et al., 2018). However, the relationship between the MTX concentration in plasma and the creatinine clearance (CCr) for children remains controversial. The MTX concentration has been reported to be negatively correlated with the CCr level Hempel et al. (2003), which agrees with the findings of this study. However, Evans et al. (1984), Joannon et al. (2004) reported there was no relationship between the MTX concentration and CCr in children. Our results showed that both the free and total MTX concentrations at 42 h were negatively correlated with CCr and that high plasma MTX levels could become a predictor of the occurrence of MTX nephrotoxicity to draw people’s attention.

### Limitations

The mainly limitation of HFCF-UF method was the NSB for some drugs. There was also no enrichment function by the HFCF-UF device when analyzing drugs at a lower concentration than the detection capability of the instrument. The MTX concentration was only monitored at 42 h for dose adjustment of leucovorin (LV) rescue which was recommended by our guidelines. It is under the steady state which should be more important to know if there is a higher free MTX. And the HFCF-UF technique has not been tested in a clinical trial. Future study should focus on overcoming these limitations. We will study the free MTX under steady state and the HFCF-UF technique will be tested in a clinical trial in our further study before it could be widely implied in clinic.

## Conclusion

We developed a simple and accurate HFCF-UF method to measure the free MTX concentration in plasma for children and successfully used the method in clinical TDM. The free MTX concentration could not be accurately estimated from the total MTX concentration at high MTX levels, cases in which toxic reactions are more likely to occur. High plasma MTX levels could become a predictor of the occurrence of MTX nephrotoxicity to draw people’s attention. The proposed HFCF-UF method is simple in that only a step of ordinary centrifugation is involved and can be used to accurately evaluate efficacy and toxicity in clinical therapeutic drug monitoring.

## Data Availability

The raw data supporting the conclusions of this article will be made available by the authors, without undue reservation.
